# An Uncommon Manifestation of Systemic Lupus Erythematosus as Lupus Enteritis With Intestinal Pseudo-Obstruction and Invasive Candidiasis

**DOI:** 10.7759/cureus.70485

**Published:** 2024-09-30

**Authors:** Rohit Patnaik, Atul Chawla, Ajay B Ramakrishnan, Nibha Jain, Negin Molazadeh, Anbalagan Pillai, Deepak S Pillai, Rajan Remya, Hardik Parmar, Nupur Karan

**Affiliations:** 1 Critical Care Medicine, Medeor 24x7 Hospital, Abu Dhabi, ARE; 2 Gastroenterology, Medeor 24x7 Hospital, Abu Dhabi, ARE; 3 Rheumatology, LLH Hospital, Abu Dhabi, ARE; 4 Cardiology, Medeor 24x7 Hospital, Abu Dhabi, ARE; 5 Orthopedics, Medeor 24x7 Hospital, Abu Dhabi, ARE; 6 Nephrology, Medeor 24x7 Hospital, Abu Dhabi, ARE; 7 Pulmonology, Medeor 24x7 Hospital, Abu Dhabi, ARE; 8 Anesthesiology, Kalinga Institute of Medical Sciences, Bhubaneswar, IND

**Keywords:** acute gastrointestinal injury, acute intestinal pseudo-obstruction, invasive candidiasis, lupus enteritis, systemic lupus erythematosus

## Abstract

Systemic lupus erythematosus (SLE) is a multifaceted autoimmune disorder, occasionally presenting with rare complications like lupus enteritis (LE) and intestinal pseudo-obstruction (IPO). We present a unique case of a 32-year-old woman with LE and IPO, complicated by invasive candidiasis, as an initial manifestation of SLE.

The patient presented with a 15-day history of abdominal pain, vomiting, and poor oral intake, and was initially misdiagnosed with infective enterocolitis. Examination revealed abdominal distension and absent bowel sounds due to IPO, alongside severe hypokalemia and signs of intra-abdominal hypertension (IAH), necessitating ventilator support. Subsequent workup confirmed SLE with LE and associated lupus nephritis (LN). The patient’s condition was further complicated by disseminated invasive candidiasis involving multiple organs, including the bloodstream, chorioretinitis, and endocarditis. Despite her critical state, intensive multidisciplinary care, including high-dose steroids, antifungal therapy, and supportive measures, led to her recovery and discharge after a 51-day ICU stay.

This case underscores the complexity of diagnosing SLE when it presents with non-specific symptoms. The concomitant occurrence of LE, IPO, and invasive candidiasis is particularly rare, highlighting the need for high clinical suspicion in the presence of SLE serological activity. The presence of invasive candidiasis was likely secondary to gut translocation due to LE-associated inflammation, a phenomenon not previously well-documented.

LE can manifest as the primary and sole presentation of SLE, even in the absence of typical lupus features. Prompt immunomodulatory treatment and comprehensive care are essential for a favorable outcome. Clinicians should consider invasive candidiasis in SLE patients with acute GI involvement, particularly in the presence of LE.

## Introduction

Systemic lupus erythematosus (SLE) is a chronic autoimmune disorder that can affect various organs, often presenting with nonspecific symptoms. Diagnosis is established by employing the criteria set forth by the American College of Rheumatology [1]. Lupus enteritis (LE), a rare complication affecting 0.2-5.8% of SLE patients, involves inflammation of the intestinal wall during periods of high disease activity [2]. LE, as described in the BILAG 2004, refers to the presence of vasculitis or inflammation in the small bowel, supported by imaging and/or biopsy data, highlighting the wide range of manifestations of the disease [2]. LE presents a non-specific clinical picture, with abdominal pain as the primary manifestation, occasionally accompanied by manifestations of impaired intestinal motility or peritonitis. Although computed tomography (CT) scanning is the gold standard for the diagnosis of LE, there is a lack of specificity of CT signs pertinent to LE. This often leads to delays in the diagnosis with subsequent complications such as bleeding, necrosis, and perforation [2]. We report an interesting case of LE and intestinal pseudo-obstruction (IPO) with invasive candidiasis by *Candida albicans* as the first presentation of SLE.

## Case presentation

History and examination 

A 32-year-old woman presented with a history of recurrent generalized abdominal pain, vomiting, loose stools, and poor oral intake for 15 days. Abdominal pain was moderate in intensity, poorly localized, and had no specific radiation. Vomiting was associated with loose stools, three to four episodes in a day, soft in consistency, not containing blood, mucus, or pus, and no history of weight loss.

The patient had no prior comorbidities; however, the patient had a history of previous hospitalization for 10 days duration in another facility, treated for infective enterocolitis with intravenous (IV) amoxicillin-clavulanate, from where the patient was referred to our facility. No history of steroid usage was present. The patient had a positive family history of SLE in two second-degree relatives from the paternal side. On physical examination, the patient was febrile, and the abdomen was tense with abdominal distension and absent bowel sounds.

Laboratory investigations

Laboratory examination showed severe hypokalemia, high white blood cell (WBC) counts, and C-reactive protein (CRP) levels (Table 1). The patient's condition quickly deteriorated due to the development of intra-abdominal hypertension (IAH), diagnosed by intra-abdominal pressure (IAP) monitoring, and the necessity for ventilator support. The patient was given IV potassium supplementation, bowel rest with no oral alimentation, and a nasogastric tube for gastric decompression. Due to the persistence of abdominal signs and symptoms, a work-up for SLE was sent, which came to be positive. The patient had a positive anti-nuclear antibody (ANA) by enzyme-linked immunosorbent assay, a negative anti-double-stranded DNA (anti-ds DNA) antibody, a low serum complement level, a positive anti-nucleosome antibody and a positive anti-Smith antibody, and a positive direct coombs test (DCT), along with anemia (Table 2). The rest of the autoimmune profile, including antineutrophil cytoplasmic antibody (c-ANCA), perinuclear anti-neutrophil cytoplasmic antibody (p-ANCA), anti-cardiolipin antibody, anti-phospholipid (APLA) antibody, and lupus anticoagulant, was negative. The patient was seronegative for human immunodeficiency virus (HIV) and hepatitis B and C. Cytomegalovirus (CMV) PCR test was negative. No evidence was found indicating the administration of oral or IV steroids, hydroxychloroquine, or other disease-modifying anti-rheumatic drugs (DMARDs). Initial two sets of blood cultures, sent on day 1 of ICU admission, and subsequent two sets of blood cultures, sent on day 6 of ICU admission, showed growth of *Candida albicans*, which was sensitive to fluconazole, caspofungin, and amphotericin B.

**Table 1:** Initial laboratory parameters SGPT, serum glutamate pyruvate transaminase; ALT, alanine transaminase; SGOT, serum glutamic-oxaloacetic transaminase; AST, aspartate transaminase; GGT, gamma glutamyl transpeptidase; ALP, alkaline phosphatase; WBC, white blood cell; CRP, C-reactive protein; BAL, bronchoalveolar lavage; AFB, acid fast bacilli

Lab parameter	Reference range (units)	Value
Hemoglobin	11.5-15.5 (g/dL)	10.9
Creatinine	44-90 (µmol/L)	44
Potassium	3.7-5.5 (mmol/L)	2.7
Total bilirubin	0.0-1.5 (mg/dL)	0.8
Direct bilirubin	<0.3 (mg/dL)	0.2
SGOT (AST)	7-38 (U/L)	30
SGPT (ALT)	13-35 (U/L)	37
GGT	5-40 (U/L)	16
ALP	32-117 (U/L)	50
WBC	3.70-11.0 (k/uL)	5.94
CRP	0-5 (mg/dL)	1.8
Procalcitonin	<0.5 (ng/mL)	0.05
Urine proteinuria	Nil	3+
Blood culture and sensitivity	-	*Candida albicans* (sensitive to fluconazole, caspofungin, and amphotericin B)
Urine culture and sensitivity	-	No growth
*Salmonella typhi* antigen O	Negative: <1:80; positive: ≥1:80	<1:20
*Salmonella typhi *antigen H	Negative: <1:160; positive: ≥1:160	<1:20
*Salmonella paratyphi* AH	Negative: <1:160; positive: ≥1:160	<1:20
*Salmonella paratyphi* BH	Negative: <1:160; positive: ≥1:160	<1:20
*Clostridioides difficile* toxin A, toxin B	-	Not detected
BAL AFB	-	Not detected

**Table 2:** Autoimmune profile parameters ANA, anti-nuclear antibody; anti-ds DNA, anti-double-stranded DNA

Autoimmune profile parameter	Reference range	Value
ANA	-	1:2560, nuclear, fine speckled
Anti-ds DNA	Negative: <4 IU/mL; indeterminate: 5-9 IU/mL; positive: ≥10 IU/mL	2 IU/mL
Serum complement C3	0.9-1.8 g/L	0.29 g/L
Serum complement C4	0.1-0.4 g/L	0.09 g/L
Anti-nucleosome antibody	Negative: <1.0; positive: ≥1.0	6.2
Anti-smith antibody	Negative: <1.0; positive: ≥1.0	2.1
Anti-cardiolipin IgM	Negative: <20 U/mL; low positive: 20-40 U/mL; medium positive: 40-80 U/mL; high positive: >80 U/mL	<1.5 U/mL
Anti-cardiolipin IgG	Negative: <20 U/mL; low positive: 20-40 U/mL; medium positive: 40-80 U/mL; high positive: >80 U/mL	<1.6 U/mL
Anti ß2 glycoprotein 1 IgM	Negative: <20 RU/mL; positive: ≥20 RU/mL	<0.8 RU/mL
Anti ß2 glycoprotein 1 IgG	Negative: <20 RU/mL; positive: ≥ 20 RU/mL	2.88 RU/mL
Lupus anticoagulant ratio (DRVV LA screen/LA confirm ratio)	0.8-1.2	1.08

Endoscopic and radiological investigations

**Figure 1:**
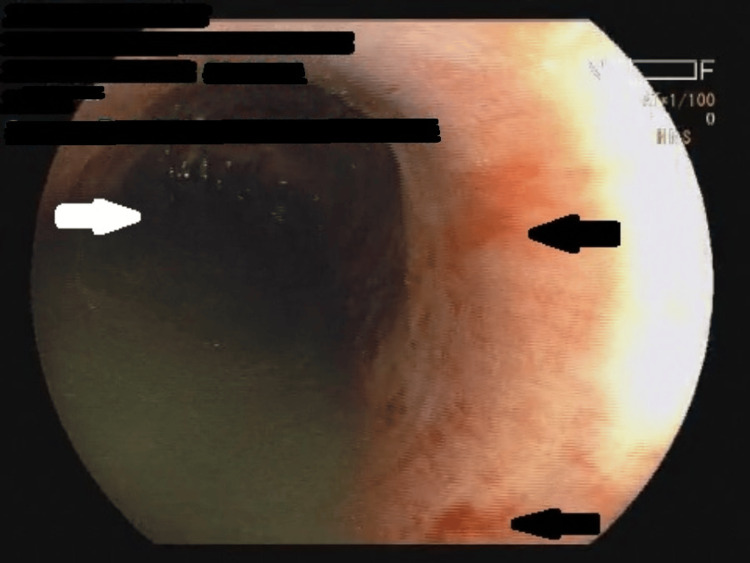
Upper GI endoscopy showing hemorrhagic erosions (black arrows) with residue (white arrow) along with dilatation of the esophagus

**Figure 2:**
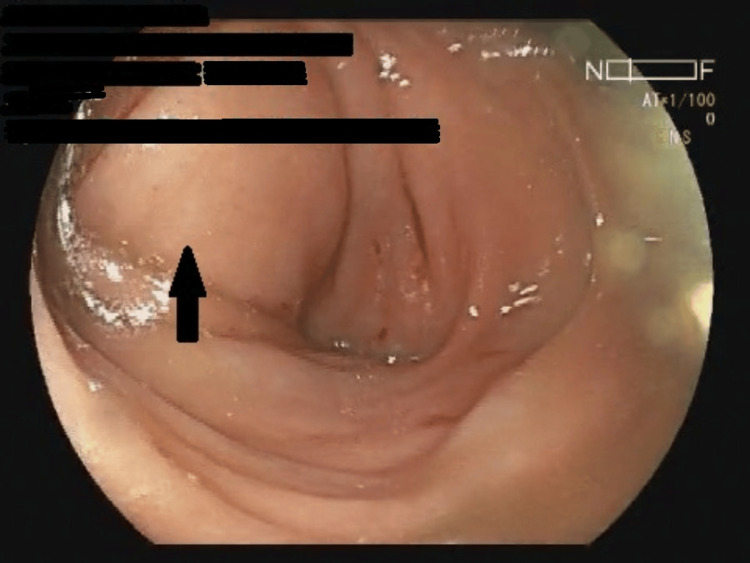
Colonoscopy showing intussusception at splenic flexure (black arrow)

**Figure 3:**
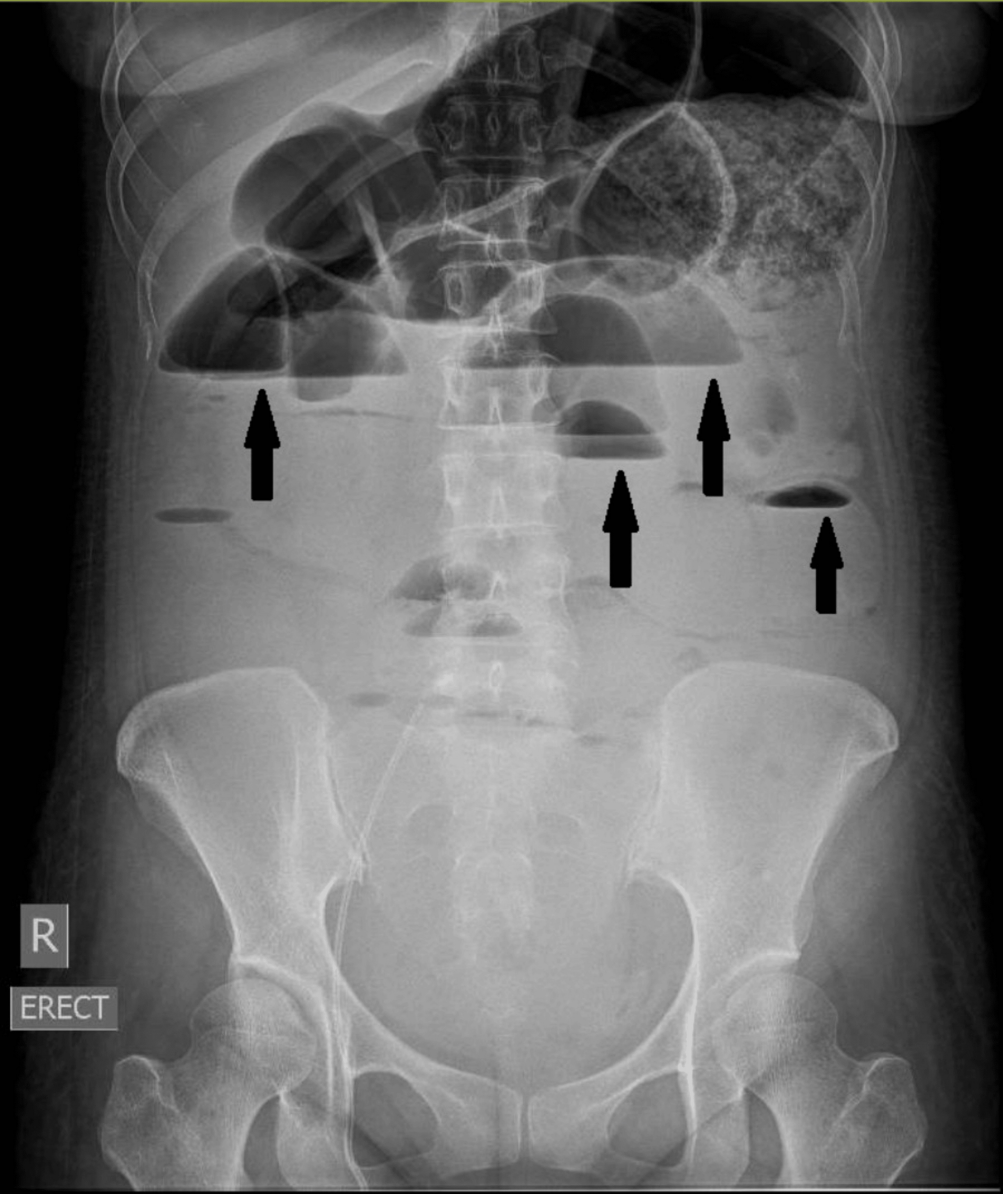
Abdominal radiograph showing air-fluid levels (black arrows) representative of IPO IPO, intestinal pseudo-obstruction

**Figure 4:**
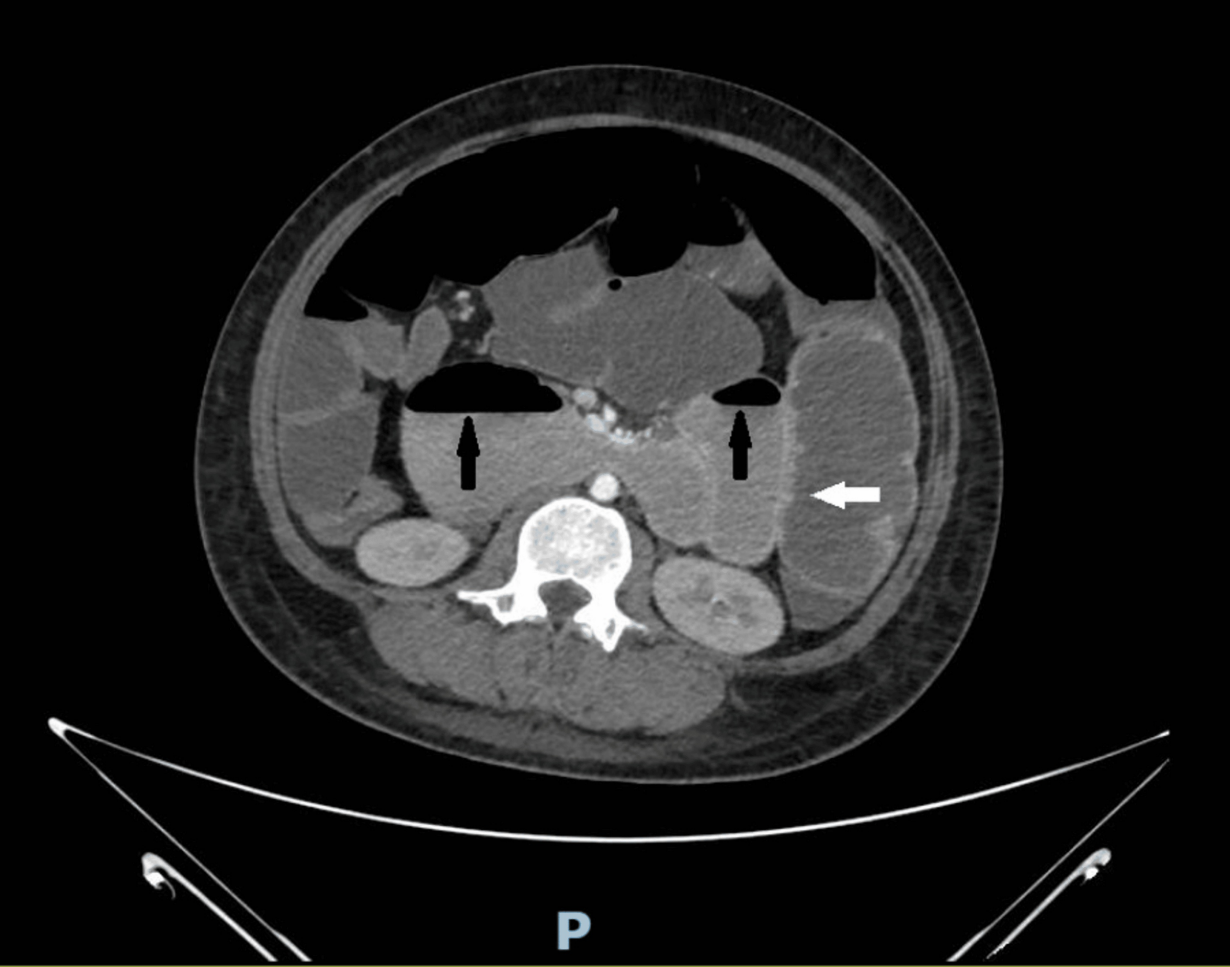
CECT of the abdomen (transverse section) showing air-fluid levels (black arrows) and bowel wall thickening (white arrow) CECT, contrast-enhanced computed tomography

**Figure 5:**
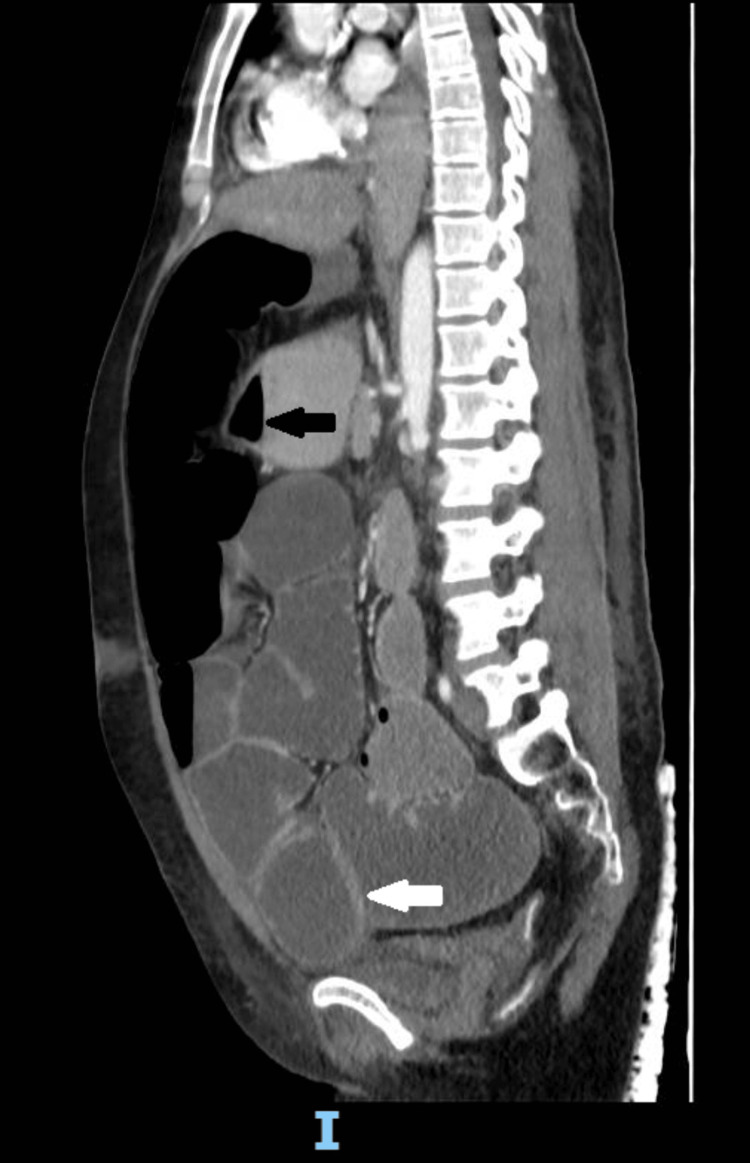
CECT of the abdomen (sagittal section) showing air-fluid levels (black arrows) and bowel wall thickening (white arrow) CECT, contrast-enhanced computed tomography

Upper GI endoscopy and colonoscopy showed severe esophagitis and a fully dilated upper GI system (Figures 1, 2). Colonic and rectal biopsies did not show any features of vasculitis. Abdominal X-rays showed dilated bowel loops with multiple air-fluid levels (Figure 3). Contrast-enhanced computed tomography (CECT) abdomen scan showed multiple areas of bowel wall thickening in the jejunum, ileum, and ascending colon, with dilated bowel loops, without any features of vasculitis (Figures 4, 5). Workup for enteric fever, tuberculosis, and *Clostridium difficile* toxin assay was negative.

Management and complications

The patient was diagnosed with SLE and LE and was subsequently started on IV hydrocortisone 200 mg/day. The patient was also found to have LN as her urine analysis showed the presence of microscopic hematuria and increased RBCs along with new-onset acute kidney injury (AKI). The CECT abdomen done had ruled out hydronephrosis or obstructive cause of AKI. In view of her critical condition, a kidney biopsy was deferred.

**Figure 6:**
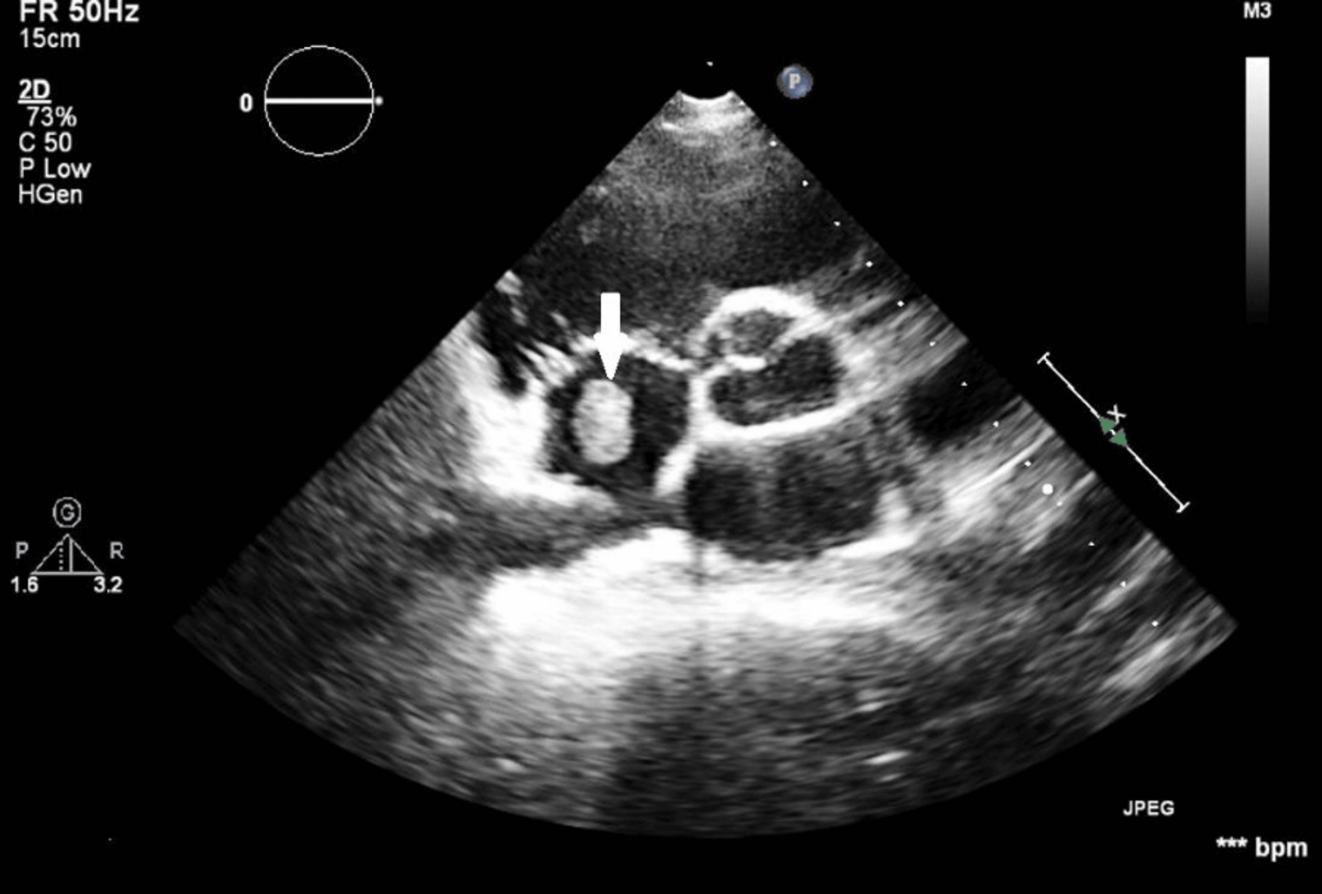
TTE in the short-axis view showing a round-shaped mass (white arrow) within the right atrium cavity. The mass measured 25x30 mm and is defined with a homogenous echotexture, suggestive of a possible thrombotic formation TTE, transthoracic echocardiogram

**Figure 7:**
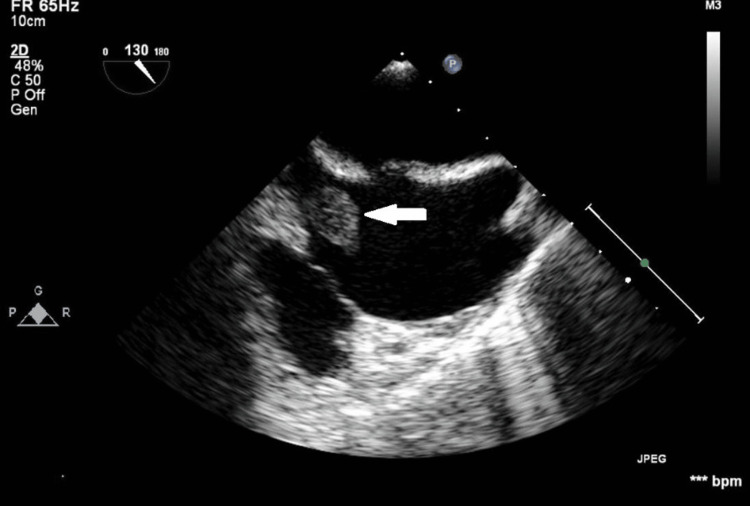
TEE view at approximately 115 degrees, focusing on RA showing a round-shaped mass (white arrow) TTE, transthoracic echocardiogram; RA, right atrium

**Figure 8:**
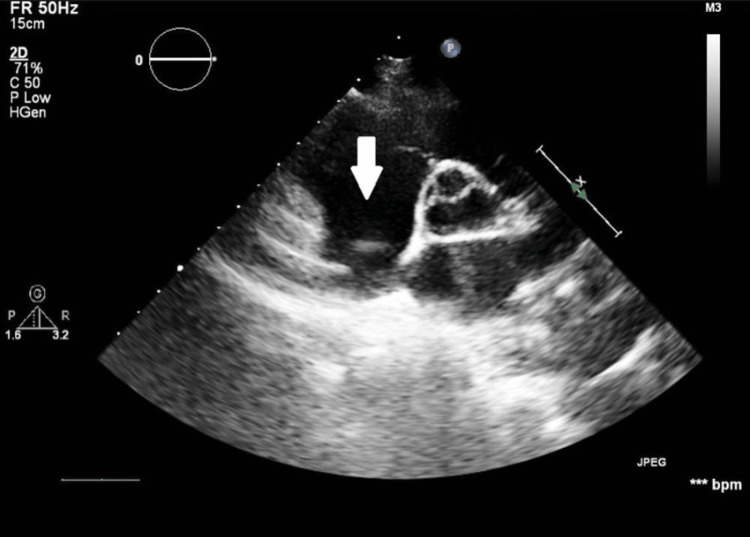
TTE in the short-axis view showing the absence of the previously observed round-shaped mass (white arrow) within the right atrium cavity TTE, transthoracic echocardiogram

Compounding the patient's condition was a diagnosis of disseminated invasive candidiasis, with involvement of multiple organs, including bloodstream candidemia, Candida chorioretinitis, and Candida endocarditis (Figures 6, 7). On day 5 of ICU admission, blood cultures showed growth of *Candida albicans*, which was sensitive to azoles, echinocandins, and amphotericin B (Table 1). On day 5 of ICU admission, an ophthalmological examination revealed Candida chorioretinitis. This necessitated prolonged antifungal therapy with IV caspofungin. On day 6 of ICU admission, a mobile mass in the right atrium near the superior vena cava (SVC), measuring 25x30 mm, was visualized on transthoracic echocardiography (TTE) and transesophageal echocardiography (TEE). On day 13 of ICU admission, this mass embolized, resulting in a pulmonary embolism, diffuse alveolar hemorrhage, and acute hypoxemic respiratory failure (Figure 8).

The patient’s clinical course was complex and challenging. The patient required extensive life support, including mechanical ventilation with prone ventilation to manage the patient's respiratory failure and acute respiratory distress syndrome (ARDS), vasopressors for fungal septic shock management, and multiple sessions of dialysis for AKI.

Outcome and follow-up

Prolonged ICU stay led to the development of delirium, which was managed through a combination of pharmacological and non-pharmacological interventions. Additionally, the patient battled critical illness neuromyopathy (CINM), which was overcome through rigorous physiotherapy and rehabilitation. After a 51-day stay in the intensive care unit, the patient was successfully discharged from the hospital with regular bowel movements on oral prednisolone 30 mg/day. The patient is on regular follow-up with no further GI symptoms.

## Discussion

The jejunum and the ileum have been reported to be the most common sites of involvement in LE [3]. LE is associated with high mortality complicated with hemorrhage, ulceration, infarction, or perforation, or if treatment or diagnosis is delayed [4].

Our patient did not have any of the typical features of SLE such as malar rash, discoid rash, alopecia, or oral ulcers. Our patient had SLE diagnosed based on positive anti-nuclear antibody along with positive anti-nucleosome and anti-smith antibodies. The presence of acute gastrointestinal injury (AGI) with bowel wall thickening on CECT abdomen with concomitant positive serological activity for lupus made us diagnose LE as the cause of the patient's abdominal signs and symptoms. We ruled out typhoid, tuberculosis, Clostridium difficile, and ischemia secondary to vasculitis. Kishimoto et al. have reported that episodes of LE can be accompanied by severe hypocomplementemia as evidenced in our case [5].

Our patient had a Systemic Lupus Erythematosus Disease Activity Index (SLEDAI) score of 23, with hypocomplementemia indicating a severe disease flare. Since GI symptoms are not given much importance in the SLEDAI score, Lin et al. have suggested that the serum complement (C3 and C4) level, rather than the SLEDAI score, be used to predict whether GI symptoms in lupus patients are caused by SLE or other non-lupus factors [6].

The presence of RBCs in urine along with AKI was supportive of LN as another manifestation of lupus flare. Supporting our diagnosis of LN was the normalization of complement levels (C3 and C4), absence of RBCs in urine, negative DCT, and resolution of abdominal signs and symptoms one week after initiation of steroid therapy.

Disseminated invasive candidiasis is a severe condition with high mortality [7]. It can affect several organ systems including blood (candidemia), eyes (Candida endophthalmitis/chorioretinitis), heart (Candida endocarditis), and viscera (hepatosplenic candidiasis and peritonitis). Our patient had candidemia, Candida chorioretinitis and Candida endocarditis.

We believe that the mobile mass in the right atrium is secondary to Candida endocarditis, because of the size and position of the mass and also the persistence of Candida albicans in blood culture. Libman-Sacks (LS) endocarditis due to SLE was less likely, as it most commonly involves basal and mid-portion of the mitral and aortic valves. We preferred medical management of this right atrial mass because of the critical condition of the patient, it was small, and it had a risk of embolization. However, subsequent embolization of the mass led to pulmonary embolism, underscoring the importance of echocardiography in SLE patients to detect asymptomatic cardiovascular involvement. 

Although SLE patients have a predisposition to invasive fungal infections (IFI), to our knowledge, this is the first case of LE as the initial manifestation of SLE presenting with invasive candidiasis. We believe that the development of invasive candidiasis could have been contributed by translocation from the gut to the bloodstream secondary to gut inflammation associated with LE [8]. Gut fungal mycobiome-host interplay has been proposed in the immunopathogenesis of SLE [8,9]. It is noteworthy that our patient did not have any of the predisposing characteristics for developing invasive candidiasis, save for a history of admission in the ICU at a prior hospital. No history of central venous catheter, total parenteral nutrition, abdominal surgery or high dose, or broad-spectrum antibiotic use was present.

Naeem et al. have also reported a similar case of LE accompanied by IPO as an initial presentation of SLE [10]. However, their case had a three-month history of fever with clinical manifestations typical of SLE such as alopecia and photosensitivity along with features of weight loss, diarrhea, and vomiting. In contrast, our case had a much shorter history of 15 days duration and without any clinical manifestations typical of SLE. Second, their case also had associated hydroureteronephrosis. Whereas, our case had disseminated invasive candidiasis, which led to a further challenge of diagnosing the background disease of LE. 

IFI in SLE most commonly affects the lungs. Most of the reported cases involved younger women who were undergoing steroid medication [11,12]. Our case is also unique because our patient neither had lung involvement nor was undergoing steroid medication at the time of occurrence of invasive candidiasis. For lupus patients who are not taking corticosteroids, such as in our case, having a high disease activity is a substantial risk factor for IFI [13]. Based on a few case series, the estimated occurrence of IFI among patients with SLE was less than 1% [14]. Additionally, the mortality rate associated with IFI in these individuals could exceed 50%. 

Accurately assessing and managing SLE patients is a complex task, and it becomes even more arduous to swiftly diagnose and treat middle-aged women who solely appear with GI symptoms without a prior history of SLE or its associated symptoms. This case underscores the complexity and challenges in managing patients with LE complicated by IPO and invasive candidiasis with multiple organ dysfunction. Prompt identification and initiation of steroid therapy along with bowel rest, hydration, correction of dyselectrolytemia, and parenteral nutrition were crucial in managing the LE. The successful outcome in this case was achieved through multidisciplinary collaboration and effective antifungal treatment with stringent infection control measures, preventing the progression of fungal sepsis.

## Conclusions

LE can present as the primary and sole manifestation of SLE. Timely identification and administration of steroids can result in a favorable outcome. The index of suspicion of LE should be high in middle-aged female patients, especially in the absence of typical lupus-related symptoms or signs, presenting with IPO. The presence of SLE serological activity including complement (C3 and C4) levels may aid in distinguishing SLE disease flare from other conditions. One should always consider the possibility of invasive candidiasis when dealing with IPO and AGI caused by LE.
